# Economic aspects of implementing genomic evaluations in a pig sire line breeding scheme

**DOI:** 10.1186/1297-9686-45-40

**Published:** 2013-10-15

**Authors:** Thierry Tribout, Catherine Larzul, Florence Phocas

**Affiliations:** 1 INRA, UMR1313 Génétique Animale et Biologie Intégrative, F-78350, Jouy-en-Josas, France; 2 AgroParisTech, UMR1313 Génétique Animale et Biologie Intégrative, F-75231, Paris, France

## Abstract

**Background:**

Replacing pedigree-based BLUP evaluations by genomic evaluations in pig breeding schemes can result in greater selection accuracy and genetic gains, especially for traits with limited phenotypes. However, this methodological change would generate additional costs. The objective of this study was to determine whether additional expenditures would be more profitably devoted to implementing genomic evaluations or to increasing phenotyping capacity while retaining traditional evaluations.

**Methods:**

Stochastic simulation was used to simulate a population with 1050 breeding females and 50 boars that was selected for 10 years for a breeding goal with two uncorrelated traits with heritabilities of 0.4. The reference breeding scheme was based on phenotyping 13 770 candidates per year for trait 1 and 270 sibs of candidates per year for trait 2, with selection based on pedigree-based BLUP estimated breeding values. Increased expenditures were allocated to either increasing the phenotyping capacity for trait 2 while maintaining traditional evaluations, or to implementing genomic selection. The genomic scheme was based on two training populations: one for trait 2, consisting of phenotyped sibs of the candidates whose number increased from 1000 to 3430 over time, and one for trait 1, consisting of the selection candidates. Several genomic scenarios were tested, where the size of the training population for trait 1, and the number of genotyped candidates pre-selected based on their parental estimated breeding value, varied.

**Results:**

Both approaches resulted in higher genetic trends for the population breeding goal and lower rates of inbreeding compared to the reference scheme. However, even a very marked increase in phenotyping capacity for trait 2 could not match improvements achieved with genomic selection when the number of genotyped candidates was large. Genotyping just a limited number of pre-selected candidates significantly reduced the extra costs, while preserving most of the benefits in terms of genetic trends and inbreeding. Implementing genomic evaluations was the most efficient approach when major expenditure was possible, whereas increasing phenotypes was preferable when limited resources were available.

**Conclusions:**

Economic decisions on implementing genomic evaluations in a pig nucleus population must take account of population characteristics, phenotyping and genotyping costs, and available funds.

## Background

In recent years, genomic selection (GS) [1] has been implemented with success in dairy cattle [2], which has made it possible to stop lengthy and costly progeny testing in this species. Compared with traditional selection schemes, GS in dairy cattle enables earlier selection from a larger number of candidates, while maintaining (for males) or even increasing (for females) the accuracy of their estimated breeding values (EBV). The resulting shorter generation intervals and higher selection intensities are expected to result in markedly higher annual genetic trends (e.g. [3]). Moreover, the savings achieved from not rearing non-productive males while awaiting the phenotype of their daughters under the progeny testing system provides the funds for genotyping. Consequently, switching from traditional to more efficient genomic schemes has not generated much, if any, additional costs in dairy cattle.

In pigs, with the availability of the porcineSNP60 Illumina beadchip (Illumina, Inc., San Diego, CA) and the extent of linkage disequilibrium (LD) observed in the nucleus populations [4,5], it is now possible to consider the implementation of GS. Current pig breeding schemes are, however, already characterized by high selection intensities and very short generation intervals. The impact of GS on these two parameters is therefore expected to be small, in contrast to the situation in dairy cattle. The accuracy of EBV is, nevertheless, generally poor in pigs, especially for late-recorded sex-limited traits and traits that cannot be measured on candidates (e.g. meat quality) or that are too expensive to measure on a large number of animals (e.g. feed efficiency). In this context, genomic evaluations can produce more accurate EBV than the current pedigree-based BLUP (best linear unbiased prediction)-animal model (pBLUP) evaluations and increase the efficiency of breeding schemes, as reported by Lillehammer et al. [6] in dam lines for traits that are only recorded on females. For sire lines, Tribout et al. [7] estimated that replacing pBLUP evaluations by genomic evaluations in a breeding scheme based on the combined phenotyping of candidates and a limited number of sibs of the candidates could increase the annual genetic trend for the population breeding goal by approximately 30% through greater accuracy, while substantially reducing the rate of inbreeding.

Most current pig breeding schemes are based on on-farm phenotyping of candidates for a few, easy to record growth and fattening traits, and on phenotyping of a limited number of sibs or candidates in testing stations for traits that are expensive or difficult to measure. Unlike in dairy cattle, GS in pigs would not result in organisational changes to the schemes that could generate sufficient savings to compensate for the cost of genotyping. First, selection among candidates usually occurs at the end of the fattening period (at about 160 days of age), which means that there is currently no financial cost associated with maintaining selection candidates during an unproductive period of time, as opposed to a breeding scheme based on progeny testing. Moreover, even if genomic evaluations no longer require candidates to be tested on-farm, the current phenotyping of candidates (mostly weighing and ultrasonic depth measurements) is much less expensive than genotyping. Finally, phenotyping of animals in testing stations for traits that are expensive or difficult to measure would still be necessary in genomic schemes in order to constitute and update the training population (TP) for these traits. Therefore, implementation of genomic evaluations would necessarily result in additional costs for pig schemes and comparing the efficiency, in terms of selection accuracy, genetic trends and inbreeding rates, of a traditional breeding scheme based on pBLUP genetic evaluation with that of a more expensive genomic breeding scheme would be clearly unfair. Thus, it is legitimate to question whether it might not be more profitable to invest in improvements in the efficiency of current schemes rather than in the implementation of genomic evaluation.

The objective of this study was to compare the efficiency – in terms of annual genetic trends and annual increases in inbreeding – of a traditional pig sire line breeding scheme based on pBLUP genetic evaluation and the combined phenotyping of candidates and their sibs, with that of a genomic breeding scheme involving the same overall cost. The main weakness of the traditional pig breeding scheme considered here is the limited number of sibs that are phenotyped per year, resulting in poor accuracy of EBV for traits that are not directly measured on the selection candidates. Thus, as a first step, we evaluated the benefits of gradually increasing the annual number of phenotyped sibs under a pBLUP scheme. The additional cost of a genomic scheme depends directly on the number of genotyped animals. Thus, in a second step, we estimated the efficiency of GS designs for different numbers of genotyped candidates. Finally, increases in the capacity to phenotype sibs and the different numbers of genotyped candidates were converted into extra annual costs compared to a reference situation that was assumed to be representative of a current standard pig sire line breeding scheme, in order to determine the most profitable investment from a genetic point of view.

## Methods

No ethical approval was required for this study because no animals were used.

### Simulated population

To compare the efficiency of different breeding schemes, we carried out a stochastic simulation of a purebred paternal pig population consisting of 1050 breeding females distributed across five herds of equal size, and 50 breeding males. The population was selected for a global breeding goal that included two genetically independent traits with an initial heritability of 0.4 and an initial genetic variance of 1, based on either traditional pBLUP EBV or genomic EBV (hereinafter referred to as PEBV and GEBV, respectively). Trait 1 was representative of a fattening trait that is inexpensive and easy to measure on-farm, and was recorded on a large number of candidates, whereas trait 2 was representative of a trait that is too difficult or too expensive to measure on a large scale (e.g. feed efficiency) or even that can not be measured on candidates (e.g. carcass composition or meat quality) and was therefore recorded on a limited number of sibs of the selection candidates. Two series of simulations were performed, considering two alternative sets of economic values in the breeding goal, i.e. W1:1 and W1:3. For W1:1, the breeding goal was $\frac{1}{\sqrt{2}}\mathrm{TB}V_{1}+\frac{1}{\sqrt{2}}\mathrm{TB}V_{2}$, whereas for W1:3, the breeding goal was $\frac{1}{\sqrt{10}}\mathrm{TB}V_{1}+\frac{3}{\sqrt{10}}\mathrm{TB}V_{2}$, where *TBV*_1_ and *TBV*_2_ are the true breeding values for traits 1 and 2, respectively. For both W1:1 and W1:3, the initial genetic variance of the breeding goal was equal to 1. In the following, these same economic weights were applied to the PEBV of animal i for traits 1 and 2 (*PEBV*_1__*i*_ and *PEBV*_2__*i*_, respectively) or to its GEBV for traits 1 and 2 (*GEBV*_1__*i*_ and *GEBV*_2__*i*_, respectively) to compute its EBV for the breeding goal in the pBLUP (*PEBV*_*gl*__*i*_) and genomic (*GEBV*_*gl*__*i*_) selection schemes.

The procedures used to simulate the genome are detailed in [7]. The simulated genome consisted of 10 pairs of 100 cM chromosomes, each chromosome carrying 1500 neutral single nucleotide polymorphism (SNP) markers, 90 biallelic randomly positioned quantitative trait loci (QTL) for trait 1, and 90 biallelic randomly positioned QTL for trait 2. All markers and QTL had a minor allele frequency higher than 0.05. The SNPs were unevenly spaced, with an average distance between two consecutive SNPs of 67 kb. Short- and long-range LD in the simulated population was comparable to the LD actually observed in the major French pig nucleus populations. By convention, allele 1 of each QTL had no effect and the absolute values of the effect of QTL allele 2 were sampled from a gamma distribution with shape and scale parameters equal to 0.4 and 1/1.66 [8], and given a positive or negative sign with probability 0.5. For both traits, the effects of the QTL were rescaled to result in a genetic variance of 1 in the breeding population when selection started. The true breeding value of animal *i* for trait *t* (*t* = 1 or 2) was calculated as: $\mathrm{TB}V_{t_{i}}=\sum_{j=1}^{10}\left(\sum_{k=1}^{90}m_{t_{\mathrm{jki}}}*q_{t_{\mathrm{jk}}}\right)$, where *m*_*tjki*_ is the genotype (i.e. 0, 1 or 2 copies of allele 2) of animal *i* for the *k*^th^ QTL for trait t on chromosome *j*, and *q*_*tjk*_ is the effect of the *k*^th^ QTL for trait *t* on chromosome *j*.

A total of 100 independent replicates were performed for each of the scenarios considered. For each replicate, a new genome was simulated and 1050 base females were randomly assigned to five herds of equal size, and within each herd into seven batches of 30 females. The time step (TS) was three weeks, which corresponded to the period between the farrowing of females from two consecutive batches. Therefore, a sow farrowed every 7 TS until culling. For simplicity, we considered that 18 TS (i.e. 378 days) corresponded to one year of simulation.

The population was first selected and bred for five years (i.e. 90 TS) under a pBLUP scenario that was designed to be representative of a traditional pig sire line breeding scheme, called BL_ref hereinafter. After this boot period, selection continued for 10 more years (i.e. 180 TS), under either the same BL_ref or according to four other pBLUP scenarios with greater capacity to phenotype sibs for trait 2, or under five genomic scenarios that differed in the annual number of candidates genotyped and the size of the TP for trait 1. The results of these alternative scenarios were compared over this 10-year period.

### Breeding scenarios

**Figure 1:**
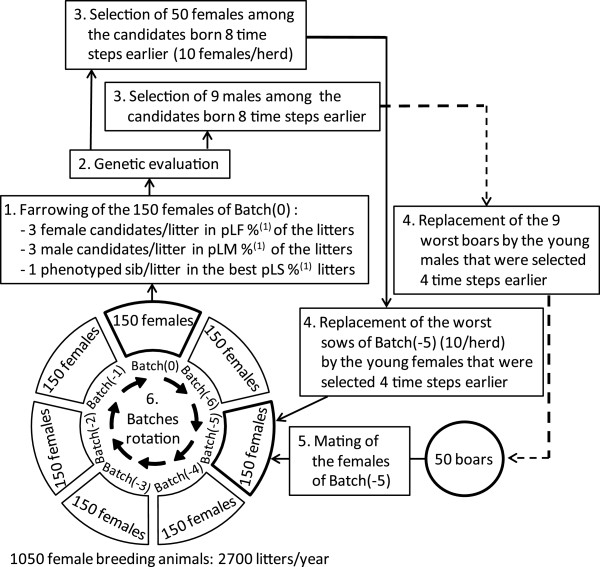
**Population structure and chronological order of events occurring at each time step. **^(1)^ pLF = proportion of litters containing three female candidates; pLM = proportion of litters containing three male candidates; pLS = proportion of litters with a sib phenotyped for trait 2 (see Table 1 for the corresponding values in the simulated scenarios).

The characteristics of the ten scenarios that were considered are summarized in Table 1 and presented in Figure 1. The numbers of boars and sows and the numbers of candidates selected per TS to replace the breeding animals was the same for each scenario.

**Table 1:** Characteristics of the simulated pBLUP and genomic breeding scenarios

	**Breeding scenarios**^**1**^									
	**BL_ref**	**BL_30%**	**BL_50%**	**BL_70%**	**BL_90%**	**GE_ref**	**GE_80%**	**GE_60%**	**GE_40%**	**GE_20%**
Proportion of litters with 3 female candidates *(pLF)*	90%	90%	90%	90%	90%	90%	73%	53%	37%	17%
Nb of litters with female candidates/herd*batch *(nLF)*	27	27	27	27	27	27	22	16	11	5
Total annual^2^ nb of female candidates, all herds together *(nFC)*	7290	7290	7290	7290	7290	7290	5940	4320	2970	1350
Proportion of litters with 3 male candidates *(pLM)*	80%	80%	80%	80%	80%	80%	63%	47%	33%	17%
Nb of litters with male candidates/herd*batch *(nLM)*	24	24	24	24	24	24	19	14	10	5
Total annual^2^ nb of male candidates, all herds together *(nMC)*	6480	6480	6480	6480	6480	6480	5130	3780	2700	1350
Proportion of litters with 1 phenotyped sib *(pLS)*	10%	30%	50%	70%	90%	10%	10%	10%	10%	10%
Nb of litters with 1 phenotyped sib/herd*batch *(nLS)*	3	9	15	21	27	3	3	3	3	3
Total annual^2^ nb of phenotyped sibs, all herds together *(nPS)*	270	810	1350	1890	2430	270	270	270	270	270
Initial size of the training population for trait 1 (at year 6) *(S1*_*0*_*)*	not relevant					13 770	10 710	8415	5355	3060
Nb of time steps to constitute the initial training population for trait 1						18	14	11	7	4
Annual^2^ increase in size of the training population for trait 1						13 770	11 070	8100	5670	2700
Initial size of the training population for trait 2 (at year 6) *(S2*_*0*_*)*	not relevant					1000	1000	1000	1000	1000
Nb of time steps to constitute the initial training population for trait 2						67	67	67	67	67
Annual^2^ increase in size of the training population for trait 2						270	270	270	270	270

^1^BL_ref, BL_30%, BL_50%, BL_70%, BL_90% = breeding schemes based on traditional pBLUP genetic evaluations using phenotypes of the candidates for trait 1 and phenotypes of sibs for trait 2 sampled in 10%, 30%, 50%, 70% or 90% of the litters, respectively; GE_ref = breeding scheme based on genomic evaluations with annual numbers of candidates and phenotyped sibs identical to BL_ref scenario; GE_80%, GE_60%, GE_40%, GE_20% = breeding schemes based on genomic evaluations with initial training population for trait 1 and annual number of candidates reduced by 20%, 40%, 60% and 80%, respectively, compared to the GE_ref scenario; ^2^the actual period of time corresponds to 18TS, i.e. 378 days, assimilated for simplicity to one year.

#### Reference pBLUP breeding scenario (BL_ref)

The BL_ref scenario is identical to the so-called “BLUP-AM” scenario described in [7] and was modelled based on the average statistics of the French Piétrain nucleus population that were obtained from the data used for national genetic evaluations. Briefly, 27 and 24 litters of the 30 litters born in each batch and on each herd (i.e. 90% and 80% of the born litters, respectively) contained three female and three male selection candidates, respectively. A phenotype for trait 1 was simulated for each candidate. A total of 15 litters per batch across the five herds (representing 10% of the born litters) also had one piglet that was phenotyped for trait 2 (these animals will be referred to as the “phenotyped sibs” in the following). These 15 litters were chosen among those containing candidates, by selecting the best litters as a function of their parental PEBV for the population breeding goal, giving priority to litters of boars with the fewest offspring among the already chosen phenotyped sibs and balancing the number of litters chosen from each herd. At each TS, the PEBV were estimated independently for the two traits, using a standard BLUP-Animal Model procedure [9] and the BLUPF90 software (http://nce.ads.uga.edu/~ignacy/newprograms.html), considering all phenotypes and pedigrees recorded since the creation of the herds. At each TS, the best 10 of the 81 female candidates from each herd and the 9 best of the 360 male candidates from across the five herds were selected based on PEBV_gl_ to partially replace the pool of male and female reproducers.

#### Alternative pBLUP scenarios

The main weakness of the BL_ref scheme lies in the limited number of sibs that are phenotyped for trait 2 (one animal in 10% of the litters), resulting in low accuracies of PEBV for candidates for this trait. Therefore, increasing the phenotyping capacity for trait 2 is expected to improve the efficiency of the traditional scheme. To evaluate this improvement, the BL_ref scenario was modified by increasing from the 90^th^ TS (that will be referred to as TS90 in the following) the proportion of litters with a phenotyped sib to 30%, 50%, 70% or 90% in the alternative BL_30%, BL_50%, BL_70% and BL_90% scenarios, respectively. Similar to the BL_ref scenario, the litters were chosen based on parental PEBV for the population breeding goal, giving priority to the litters of boars with the fewest offspring among the already chosen phenotyped sibs and balancing the number of litters chosen from each herd. BL_90% corresponded to the case where all litters that had female candidates (i.e. 90% of the born litters) also had one sib that was phenotyped for trait 2 (i.e. no litter selection).

#### Reference genomic breeding scenario (GE_ref)

The GE_ref scenario differed from the BL_ref scenario only by the procedure used to estimate breeding values; the numbers of male and female candidates, phenotyped sibs, selected candidates and breeding males and females were the same. This scenario was similar to the “GE-2TP” scenario detailed in [7].

Starting in TS90, all candidates, phenotyped sibs and breeding animals of the GE_ref scenario were genotyped, and all selection and culling decisions were based on *GEBV*_*gl*_, which were estimated as follows. At TS90, the effects of all SNPs on traits 1 and 2 were estimated using the BLUP methodology described in [1] on two distinct TP, one for each trait. The initial TP for trait 2 (TP2) consisted of 1000 phenotyped sibs of the candidates born between TS16 and TS82, that were genotyped and phenotyped for trait 2. For trait 1, the initial TP (TP1) consisted in 13 770 male and female candidates that were genotyped and phenotyped for this trait during the 5^th^ year of the simulation (TS73 to TS90).

From a practical point of view, the genome simulated in this study was approximately three times shorter than the real pig genome (10 M instead of 30 M). Therefore, TP1 and TP2 were simulated by randomly sampling one third of the candidates and phenotyped sibs at each TS, as described and illustrated in [7], in order to produce GEBV with appropriate accuracies [10]. Thus, the initial TP1 and TP2 contained 4590 and 333 simulated animals, respectively, equivalent to 13 770 or 1000 animals, respectively, for a full genome.

The estimated SNP effects were used in the current TS and during the next 17 TS (i.e. 1 year) to calculate the GEBV of the breeding animals and candidates for trait *t* as $\mathrm{GEB}V_{t_{i}}=\sum_{j=1}^{10}\left(\sum_{k=1}^{1500}m_{t_{\mathrm{jki}}}*{\hat{g}}_{t_{\mathrm{jk}}}\right)$, where ${\hat{g}}_{t_{\mathrm{jk}}}$ is the estimated effect of the *k*^th^ SNP on chromosome *j* for trait *t*. Then, *GEBV*_1*i*_ and *GEBV*_2*i*_ were combined based on their economic weights to calculate *GEBV*_*gli*_.

Each year, the effects of the SNPs on the two traits were re-estimated using a TP that was increased by data from the 270 new phenotyped sibs and the 13 770 new candidates that were phenotyped in the past year (in fact, 90 and 4590 randomly sampled additional animals, respectively, to account for the size of the simulated genome), and these new estimates were then used to calculate the *GEBV*_*ti*_ of breeding animals and candidates for the current and next 17 TS. Thus, the size of TP2 increased from 1000 to 3430 animals from the 6^th^ to the 15^th^ year of simulation. To limit computation time, the pigs of the oldest year were removed from TP1 every year, starting in year 10. Thus, the size of TP1 increased from 13 770 animals at year 6 to 55 080 at year 9, and then remained stable. Preliminary results had shown that the loss in accuracy for trait 1 due to removal of the oldest animals from TP1 was very low (approximately 1% in year 10).

#### Alternative genomic breeding scenarios

Under the GE_ref scenario, the number of candidates that were genotyped per year from year 5 to year 15 was identical to the annual number of candidates in the BL_ref scheme (13 770 animals/year). The extra cost due to genotyping was therefore considerable in the GE_ref scenario. A reduction in the annual number of genotyped candidates would reduce this cost but also cause a loss of technical efficiency of the GS scheme because of the resulting decreases in selection intensities, size of TP1 and accuracies of GEBV for trait 1. To evaluate this loss, four genomic schemes were derived from the GE_ref scenario. Under these schemes, the initial size of TP1 (at TS90) and the number of candidates genotyped per batch from the 6^th^ year of simulation were reduced by approximately 20% (GE_80% scheme), 40% (GE_60%), 60% (GE_40%) and 80% (GE_20%), as described below:

1) Instead of all candidates from the 18 TS of the 5^th^ year under the GE_ref scenario, the initial TP1 under the GE_80%, GE_60%, GE_40% and GE_20% scenarios included male and female candidates that were phenotyped in the last 14, 11, 7 and 4 TS of year 5, respectively;

2) The numbers of litters, per herd and TS, with three male and three female candidates that were phenotyped and genotyped (nLM = 24 and nLF = 27 litters under the GE_ref scenario, respectively) were reduced to 19 and 22 litters for GE_80%, to 14 and 16 litters for GE_60%, to 10 and 11 litters for GE_40%, and to 5 and 5 litters for GE_20%. At each TS, these litters were chosen in each herd from among the 27 litters available by selecting the best ones based on parental EBV for the breeding goal.

### Technical efficiency criteria

One hundred independent replicates of five years of selection under the BL_ref scenario, followed by 10 years of selection under each of the 10 alternative scenarios, were simulated for the W1:1 and W1:3 breeding goals. For each replicate of each breeding scenario x breeding goal combination, the following summary statistics were calculated:

the mean of the true breeding values of the piglets born in each TS for traits 1 and 2 and for the breeding goal, in order to measure the realized genetic gain;

the mean of the inbreeding coefficients of the piglets born in each TS based on pedigree information using PEDIG software [11], to measure the evolution of inbreeding over time;

the correlation between the TBV and the PEBV or GEBV of the selection candidates for each TS, in order to estimate accuracies of the selection for traits 1 and 2.

The above statistics were then used to derive the average annual realized genetic gains (ΔGa), the average annual increase in inbreeding (ΔFa), and the average accuracies from year 6 to year 15 for each replicate and averaged over the 100 replicates for each breeding scenario x breeding goal combination.

### Evaluation of the extra costs of the alternative breeding schemes

To be able to compare the relative efficiencies of the genomic and pBLUP simulated schemes at constant cost, we evaluated the extra cost of the nine alternative schemes compared to the BL_ref reference scenario, as follows.

#### Alternative pBLUP schemes

We assumed that the increase in the number of phenotyped sibs would generate extra costs linked to the building of additional testing station facilities and the raising and phenotyping of animals. Based on the range of costs observed in testing stations in France and other countries (IFIP, French Pork and Pig Institute survey in 2005, personal communication), and assuming amortization of buildings and equipment over a 10-year period, a low (€150), medium (€350) and high (€550) cost per extra sib phenotyped for trait 2 were considered. The annual extra costs of the alternative pBLUP schemes, compared to the BL_ref scheme, were therefore calculated by multiplying the annual numbers of additional phenotyped sibs (540, 1080, 1620 and 2160 animals for BL_30%, BL_50%, BL_70% and BL_90%, respectively) by the individual additional cost under the low, medium and high cost hypotheses.

#### Alternative genomic schemes

Implementation of genomic selection requires genotyping of the initial TP1 and TP2, and continuous genotyping of new candidates and phenotyped sibs. To evaluate the resulting extra costs, we considered that boars and sows used for breeding were genotyped with the porcine60SNP beadchip but that selection candidates and phenotyped sibs were genotyped with a lower density and less expensive beadchip. Indeed, several studies [12-14] have shown that it is possible to impute the genotypes of young animals for the missing SNPs with a very low error rate when both parents are genotyped with the denser beadchip. Three hypotheses were considered to take uncertainties regarding the future evolution of genotyping costs into account: the genotyping costs considered for the high density (HDpr) and the low density (LDpr) chips were €150/animal and €50/animal, respectively, under the high price hypothesis, €120 and €30 under the medium price hypothesis, and €90 and €10 under the low price hypothesis.

The numbers of animals in the initial TP1 (S1_0_) under the five genomic scenarios considered are in Table 1. The sires and dams of these animals were genotyped with the denser chip to increase the imputation accuracy; for simplicity, we considered a constant number of parents regardless of the genomic scenario, equal to the annual number of new breeding animals (900 gilts and 162 boars). The S2_0_ (i.e. 1000) phenotyped sibs that constitute the initial TP2 were assumed to be genotyped with the high density chip rather than imputed. The genotyping costs for the initial TP1 and TP2 were therefore calculated as GCini = S1_0_*LDpr + (S2_0_ + 900 + 162)*HDpr. Similar to the amortization of additional testing station capacity under the pBLUP scenarios, these initial genotyping costs were spread over a 10-year period. In addition, nFC female candidates, nMC male candidates and nPS phenotyped sibs were genotyped every year for the low density chip, and the 900 female candidates and the 162 male candidates selected each year as breeding animals were re-genotyped with the denser chip to enable high density imputation of their future offspring. Thus, the annual extra cost of any of a genomic scheme was calculated as:

$$\begin{array}{l} \mathrm{aGC}=\left(\left(\mathrm{nMC}+\mathrm{nFC}+\mathrm{nPS}\right)*\mathrm{LDpr}+\left(900+162\right)*\mathrm{HDpr}\right)\\ \;+\mathrm{GCini}/10\\ =\left(\left(\mathrm{nMC}+\mathrm{nFC}+\mathrm{nPS}\right)+\left({S1}_{0}/10\right)\right)*\mathrm{LDpr}+1268.2*\mathrm{HDpr}. \end{array}$$

## Results

### Accuracies and genetic trends

#### Comparison of the pBLUP and genomic reference breeding schemes

Overall, the GE_ref breeding scheme produced higher genetic gains than the BL_ref breeding scheme. When the population was selected for the W1:1 breeding goal, GE_ref resulted in 11%, 83% and 28% higher ΔGa than BL_ref for trait 1, trait 2 and the breeding goal, respectively (Table 2). This superiority was due to more accurate EBV in the genomic scheme (Table 3). Whereas the accuracy of the PEBV of candidates was equal to only 0.62 for trait 1 and 0.33 for trait 2 on average over the considered period, the average accuracy of the GEBV increased from approximately 0.76 (year 6) to 0.85 (year 9) for trait 1, and from 0.45 (year 6) to 0.55 (year 15) for trait 2, as the sizes of TP1 and TP2 increased (results not shown). On average, over the 10-year comparison, the accuracy of the GEBV was 0.83 and 0.52 for traits 1 and 2, respectively, i.e. 34% and 58% higher than the accuracy of the PEBV. The superiority of GE_ref over BL_ref in terms of ΔGa was much greater for trait 2 than for trait 1 because the increase in accuracy was greater for trait 2 than for trait 1. This indirectly reinforced the relative importance of trait 2 compared to trait 1 in the selection index.

**Table 2:** Average annual genetic trends under different scenarios, for two breeding goals

	**Average annual genetic trends, in genetic standard deviation units**					
	**Trait 1**		**Trait 2**		**Breeding goal**	
**Relative weights on traits 1 and 2 in the breeding goal**^**(2)**^	**1:1**	**1:3**	**1:1**	**1:3**	**1:1**	**1:3**
Scenarios^(1)^						
BL_ref	0.63^a^	0.42^a^	0.18	0.31	0.57	0.43
BL_30%	0.60	0.36^c^	0.27	0.42	0.61^a^	0.51
BL_50%	0.58^b^	0.35^d^	0.32^a^	0.45	0.63^b^	0.54^a^
BL_70%	0.58^b^	0.33^de^	0.33^b^	0.48^a^	0.64^b^	0.56
BL_90%	0.57	0.33^e^	0.36	0.50^bc^	0.65	0.58^bc^
GE_ref	0.70	0.40^ab^	0.33^b^	0.51^c^	0.73	0.61
GE_80%	0.68	0.39^b^	0.34^b^	0.50^bc^	0.72	0.60^d^
GE_60%	0.66	0.37^c^	0.34^b^	0.50^b^	0.71	0.59^cd^
GE_40%	0.62^a^	0.35^de^	0.34^b^	0.49^b^	0.68	0.57^b^
GE_20%	0.54	0.29	0.33^ab^	0.48^a^	0.61^a^	0.54^a^

The results presented are means of the 100 replicates; standard deviations of the 100 replicates ranged from 0.05 to 0.07; ^1^see Table 1 for a description of the different breeding scenarios;

^2^1:1 Breeding goal = 12 Breeding Value for trait 1 + 12 Breeding value for trait 2;

1:3 Breeding goal = 110 Breeding Value for trait 1 + 310 Breeding value for trait 2; within a column: all means differ (p < 0.01) except means with common superscripts.

**Table 3:** Average accuracy of the estimated breeding values of candidates for different scenarios, for two breeding goals

	**Average accuracy of estimated breeding values for**			
	**Trait 1**		**Trait 2**	
**Relative weights on traits 1 and 2 in the breeding goal**^**(2)**^	**1:1**	**1:3**	**1:1**	**1:3**
Scenario^(1)^				
BL_ref	0.62	0.68	0.33	0.28
BL_30%	0.63	0.69^a^	0.41	0.36
BL_50%	0.63^a^	0.69^a^	0.44	0.39
BL_70%	0.63^ab^	0.69^a^	0.46	0.41
BL_90%	0.64^b^	0.69^a^	0.48	0.41
GE_ref	0.83	0.88	0.52^a^	0.47
GE_80%	0.81	0.87	0.51^a^	0.46^a^
GE_60%	0.78	0.85	0.51^a^	0.46^a^
GE_40%	0.75	0.82	0.51^a^	0.46^a^
GE_20%	0.68	0.76	0.52^a^	0.46^a^

The results presented are means of the 100 replicates; standard deviations of the 100 replicates ranged from 0.01 to 0.02 for trait 1, and from 0.02 to 0.05 for trait 2; ^1^see Table 1 for a description of the different breeding scenarios; ^2^see Table 2 for a description of the breeding goals; within a column: all means differ (p < 0.01) except for values with the same superscripts.

When the population was selected for the W1:3 breeding goal, GE_ref also resulted in markedly higher ΔGa than BL_ref for trait 2 and for the breeding goal (64% and 41%, respectively), but produced a slightly lower ΔGa for trait 1 than the traditional scheme (−5%), despite the fact that the GEBV were more accurate than the PEBV for both traits. This result was due to the greater weighting of trait 2 in the W1:3 breeding goal than in the W1:1 breeding goal. Indeed, the larger weight on trait 2 in the W1:3 breeding goal counterbalanced its lower accuracy compared to that of trait 1 in the BL_ref scenario. Therefore, the selection pressure on the two traits was more balanced for W1:3 than for W1:1, for which the selection pressure was mainly on trait 1. Because of the greater increase in accuracy for trait 2 than for trait 1 (+68% vs. +30%) when switching from BL_ref to GE_ref, the selection pressure on trait 2 in the W1:3 selection index, which was already large in BL_ref, was further increased in GE_ref. Because of the high selection pressure on trait 2, a small loss of selection response was observed for trait 1.

#### Improved pBLUP scenarios with larger numbers of phenotyped sibs

The accuracy of the PEBV of candidates for trait 2 improved significantly when the proportion of litters with one sib phenotyped for trait 2 increased (Table 3). Phenotyping one sib in 90% rather that in 10% of the litters resulted in an increase in the accuracy of the PEBV by on average 45%: 0.48 for BL_90% and 0.33 for BL_ref, for the W1:1 breeding goal. This improvement in accuracy for trait 2 was due to the larger number of half-sibs phenotyped for this trait per candidate, as well as the higher proportion of candidates that benefited from having one full-sib phenotyped. However, the average accuracy for trait 2 was lower for all pBLUP scenarios than for GE_ref. This greater accuracy for trait 2 from increasing the number of phenotyped sibs in the pBLUP schemes improved the genetic gains for this trait (Table 2) markedly. For example, testing one phenotyped sib in 50% instead of 10% of the litters resulted in a 71% greater ΔGa (+0.32σ_g_/yr vs. +0.18σ_g_/yr) with selection for the W1:1 breeding goal. In the extreme case of BL_90%, the average annual genetic trend for trait 2 (+0.50σ_g_/yr) almost equalled that of the GE_ref with the W1:3 breeding goal, and even slightly exceeded (+0.36σ_g_/yr) that of GE_ref with the W1:1 breeding goal.

As expected, accuracy of PEBV for trait 1 was the same for all pBLUP scenarios, since the number of phenotyped candidates was kept constant and the sibs were not phenotyped for trait 1. Nevertheless, it should be noted that the ΔGa for trait 1 declined with an increase in the number of sibs phenotyped for trait 2 because of the increasing selection pressure on trait 2, resulting from the increasing accuracy for trait 2. Logically, this unfavourable impact on trait 1 was greater when the population was selected for a breeding goal that placed more emphasis on trait 2. However, the genetic gain for trait 1 remained positive in all the cases.

Overall, increasing the number of phenotyped sibs significantly improved the efficiency of the pBLUP breeding scheme in terms of response in the breeding goal. The extreme BL_90% scenario, in which one animal from every litter suitable for genetic purposes was tested in station, led to a 14% increase in the ΔGa in the breeding goal with selection on W1:1, and a 35% increase with selection on W1:3. However, even in this very favourable case, the pBLUP scenario had 11% and 5% less response in the breeding goal than the GE_ref scheme with selection on W1:1 and W1:3, respectively.

#### Genomic scenarios with reduced numbers of candidates

Compared to GE_ref, reducing the initial size of TP1 and thereafter the number of litters containing genotyped candidates resulted in a lower ΔGa for trait 1 (Table 2). The loss of ΔGa for trait 1 was minor with small reductions in genotyping (approximately −3% and −7% compared to GE_ref for GE_80% and GE_60%, respectively) but was more marked with greater reductions in genotyping (−12% and −14% for GE_40%, and −24% and −29% for GE_20% compared to GE_ref for W1:1 and W1:3, respectively). The origins of these losses were twofold. First, reducing the number of genotyped candidates reduced the initial size and growth of TP1 and, therefore, reduced the accuracy of the GEBV for trait 1 (Table 3). However, the reduction in the accuracy of the GEBV for trait 1 was only moderate. For example, the average accuracy over the 10-year period for W1:1 was only 5.5% lower for GE_60% than for GE_ref, despite a 40% reduction in the size of TP1, and only 9.4% lower for GE40% than for GE_ref, despite a 60% reduction in the size of TP1, and remained greater than for the pBLUP schemes. The second cause for the reduction in ΔGa for trait 1 was a decrease in selection intensity, since a fixed number of young animals (10 females per TS in each herd, and nine males per TS) were selected from a smaller number of candidates.

The size and structure of TP2 was the same for the five genomic scenarios. Consequently, the accuracy of GEBV for trait 2 remained unchanged (Table 3). Thus, the decline in accuracy for trait 1 from GE_ref to GE_20% resulted indirectly in an increase in the selection emphasis on trait 2 in the selection index. This counterbalanced the negative effects of the lower selection intensity due to the smaller number of candidates on response for trait 2. Thus, ΔGa for trait 2 was not (for the W1:1 breeding goal) or only slightly (−6% from GE_ref to GE_20% for the W1:3 breeding goal) impacted by the reduction in the number of candidates (Table 2).

As a consequence of the impact on responses for traits 1 and 2, the efficiency of the genomic breeding scheme in improving the breeding goal was only moderately affected by the number of litters with candidates, and remained much greater than for BL_ref in all cases (Table 2). Reducing the initial size of TP1 and the number of litters with candidates by 40% and 80% only reduced the ΔGa for the breeding goal by 4% and 16%, respectively, compared to the GE_ref scenario, when the population was selected for the W1:1 breeding goal. This loss in efficiency was even less marked when a greater weight was given to trait 2 in the breeding goal (−3% and −11%, respectively, for GE_60% and GE_20% compared to GE_ref).

#### Economic comparison of genomic and pBLUP schemes

**Figure 2:**
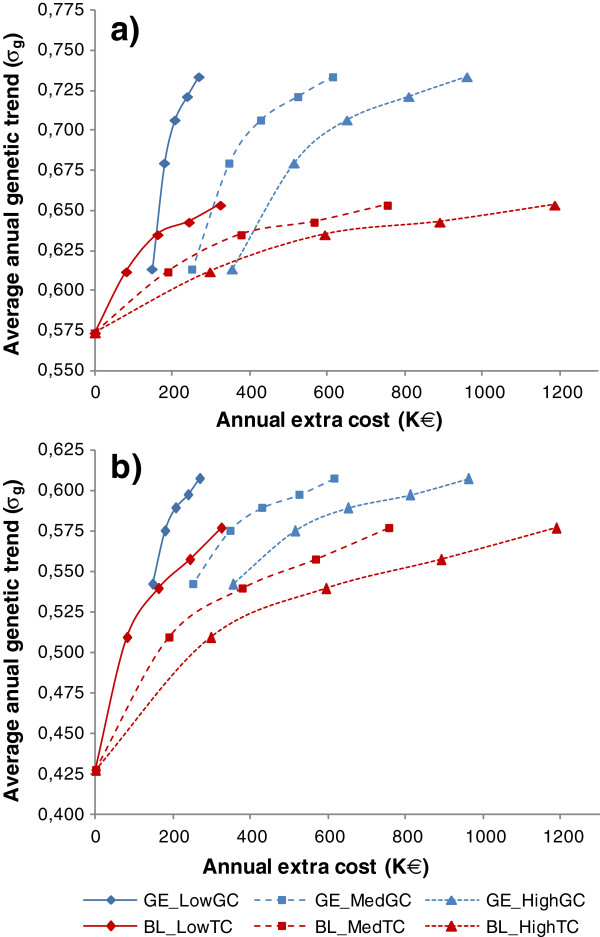
**Average annual genetic trend for the breeding goal**^**1 **^**under genomic and pBLUP scenarios, based on the additional annual cost compared to the reference scheme**^**2**^**.** The results are averages of the 100 replicates; ^1^ Breeding goal = 12 Breeding Value for trait 1 + 12 Breeding Value for trait 2 **(Figure a)** or 110 Breeding Value for trait 1 + 310 Breeding Value for trait 2 **(Figure b)**; ^2^ three levels of genotyping and phenotyping costs were considered: GE_LowGC, GE_MedGC and GE_HighGC = genomic breeding scheme under low, medium and high genotyping costs hypothesis, respectively; BL_LowTC, BL_MedTC and BL_HighTC = pBLUP breeding scheme under low, medium and high testing station costs hypothesis, respectively.

The ΔGa for the breeding goal (ΔGa_bg) produced under the five pBLUP and five genomic schemes, as a function of their additional annual costs compared to the BL_ref scheme, are plotted in Figure 2a and 2b for the W1:1 and W1:3 breeding goals, respectively. These comparisons were made under the three hypotheses for the cost of genotyping and under the three hypotheses for the cost of phenotyping additional sibs. Interpolations were performed to estimate the ΔGa_bg for numbers of litters with one phenotyped sib and for genotyping reductions that were intermediate to those simulated. The lower bound for the three pBLUP scenarios corresponded to the BL_ref scheme (no additional cost), whereas the upper bound corresponded to the BL_90% scheme, which was the most expensive and most efficient scenario in terms of ΔGa_bg. Similarly, the lower bound for the three genomic schemes was observed for the least expensive and least efficient scenario (GE_20%), whereas the upper bound corresponded to the most expensive and most efficient scenario (GE_ref).

When ignoring the extra costs, the genomic breeding schemes yielded markedly higher ΔGa_bg than the most efficient pBLUP scheme (BL_90%) when candidates from at least 30% (for W1:1) or at least 40% (for W1:3) of the best litters with candidates in the reference scheme were genotyped. The potential extra gain in ΔGa_bg between the best genomic scheme (GE_ref) and the best pBLUP scheme (BL_90%) was, however, greater for the W1:1 breeding goal (ΔGa = 0.73σ_g_/yr vs. 0.65σ_g_/yr, i.e. an increase of 12%) than for the W1:3 breeding goal (ΔGa = 0.61σ_g_/yr vs. 0.58σ_g_/yr, i.e. an increase of 5%). Conversely, the traditional schemes always resulted in lower ΔGa_bg than the least efficient genomic scheme (GE_20%) when fewer than 30% (in the W1:1 case) or 50% (in the W1:3 case) of the litters had a sib phenotyped for trait 2.

Implementing the cheapest possible genomic scheme considered in this study (GE_20%) obviously involved a minimum extra annual cost, depending on the cost of genotyping an individual. For example, under the medium genotyping cost hypothesis, this minimum investment amounted to €250 000 per year. Below this threshold for annual additional expenses, the only possibility to improve the efficiency of the breeding programme would be to increase the number of phenotyped sibs.

Comparing the respective ΔGa_bg resulting from the pBLUP scheme and from the genomic scheme that could be implemented at a given extra cost, revealed two areas where the most efficient of the two strategies differed. Below a certain threshold for extra annual costs, ΔGa_bg was greater for the improved pBLUP scheme than for the genomic scheme, and above this threshold, the genomic scheme was more efficient. The value of this threshold for each combination of genotyping costs and costs of phenotyping an additional sib is presented in Table 4. Table 4 shows that, for a given cost of phenotyping an additional sib, this threshold increased with the genotyping cost. Conversely, for a given genotyping cost, the threshold decreased as the phenotyping cost of sibs increased. To illustrate this, for W1:1, an extra annual cost of €260 000 was more effective if used to implement a genomic breeding scheme than to improve the efficiency of the pBLUP scheme when the genotyping costs were low (regardless of the phenotyping cost of sibs), or when genotyping costs were medium and phenotyping costs of sibs were high. In all other cases, these extra funds would be more efficiently used to increase the number of phenotyped sibs in the traditional scheme.

**Table 4:** Thresholds for the extra annual cost (in K€/yr) that delimit areas of interest to improve the pBLUP scheme or implement the genomic scheme, for two breeding goals

**Cost of phenotyping additional sibs**	**Relative weights on traits 1 and 2 in the breeding goal **^**(1)**^					
	**1:1**			**1:3**		
	**Genotyping cost**					
	**Low**	**Medium**	**High**	**Low**	**Medium**	**High**
Low	160	300	450	145	350	530
Medium	145	270	410	145	250	350
High	145	250	370	145	250	350

^1^See Table 2 for a description of the breeding goals; below the threshold: improving the pBLUP breeding scheme resulted in higher genetic trends for the population breeding goal than implementing genomic evaluations; above the threshold: implementing genomic evaluations resulted in higher genetic trends for the population breeding goal than improving the pBLUP breeding scheme.

### Inbreeding

Rates of inbreeding (ΔFa) observed in the 10 scenarios simulated for the two breeding goals are in Table 5.

**Table 5:** Average annual increase in inbreeding under the scenarios compared, for two breeding goals

**Scenario**^**(1)**^	**Average annual increase in inbreeding coefficient, %**	
	**Relative weights on traits 1 and 2 in the breeding goal**^**(2)**^	
	**1:1**	**1:3**
BL_ref	+0.84^a^*(0.28)*	+1.58^a^*(0.53)*
BL_30%	+0.85^a^*(0.26)*	+1.48^ab^*(0.43)*
BL_50%	+0.85^a^*(0.26)*	+1.39^bc^*(0.34)*
BL_70%	+0.86^a^*(0.27)*	+1.32^c^*(0.33)*
BL_90%	+0.88^a^*(0.22)*	+1.41^bc^*(0.40)*
GE_ref	+0.36^b^*(0.13)*	+0.51^d^*(0.23)*
GE_80%	+0.39^b^*(0.13)*	+0.52^d^*(0.20)*
GE_60%	+0.46 *(0.16)*	+0.58 *(0.21)*
GE_40%	+0.56 *(0.16)*	+0.65 *(0.20)*
GE_20%	+0.76 *(0.22)*	+0.84 *(0.25)*

The results presented are means and (*standard deviations)* of the 100 replicates; ^1^see Table 1 for a description of the different breeding scenarios; ^2^see Table 2 for a description of the breeding goals; within a column: all means differ (p < 0.01) except for values with the same superscripts.

#### Comparison of the pBLUP and genomic reference breeding schemes

**Figure 3:**
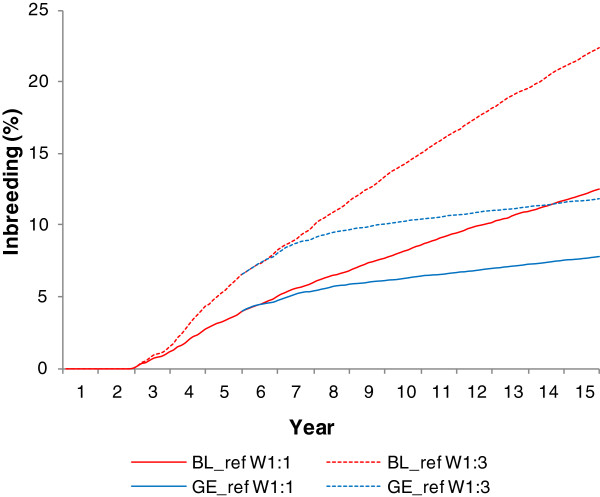
**Evolution of population inbreeding under the pBLUP and genomic reference scenarios**^**1**^**, for two breeding goals**^**2**^**.** Averages of the 100 replicates; ^1^simulated scenarios: BL_ref = breeding scheme based on traditional pBLUP genetic evaluations using phenotypes of the candidates for trait 1 and phenotypes of sibs for trait 2 sampled in 10% of the litters; GE_ref = breeding scheme based on genomic evaluations with annual numbers of candidates and phenotyped sibs identical to the BL_ref scenario; ^2^ W1:1: Breeding goal = 12 Breeding Value for trait 1 + 12 Breeding Value for trait 2; W1:3: Breeding goal = 110 Breeding Value for trait 1 + 310 Breeding Value for trait 2.

The GE_ref scheme resulted in a smaller increase in the average inbreeding coefficient of the population than the BL_ref scheme, for both W1:1 and W1:3 (Figure 3). For GE_ref, following implementation of genomic evaluations, the rate of increase in inbreeding initially gradually declined, reflecting the progressive replacement of breeding animals selected based on PEBV until TS90 by males and females selected based on GEBV. Thereafter, the average population inbreeding rose at a steady rate that was similar for W1:1 and W1:3 but significantly lower than for BL_ref. Considering the complete 10-year period, ΔFa was on average 57% lower for GE_ref than for BL_ref when the population was selected based on the W1:1 breeding goal (+0.36% per year vs. +0.84% per year) and 68% lower for the W1:3 breeding goal (+0.51% per year vs. +1.58% per year).

#### Improved pBLUP scenarios with larger numbers of phenotyped sibs

No significant relationship was found between the annual number of sibs phenotyped for trait 2 and ΔFa when the population was selected based on the W1:1 breeding goal. When greater weight was given to trait 2 in the breeding goal (W1:3), increasing the proportion of litters with a sib recorded for trait 2 from 10 to 70% significantly reduced the annual increase in inbreeding by −17%. However, ΔFa tended to be 7% higher for BL_90% than for BL_70% (*P*-value of the difference = 0.053).

#### Genomic scenarios with reduced numbers of candidates

Increasing restrictions on the number of litters with candidates logically resulted in selecting males and females that were more related to each other. This significantly accelerated the increase in inbreeding in the population. For example, the average inbreeding coefficient increased 8, 28, 55 and 111% faster for GE_80%, GE_60% GE_40% and GE_20%, respectively, than for GE_ref, when the population was selected based on the W1:1 breeding goal. However, the global increase in inbreeding over the 10-year period was smaller for all genomic schemes than for any of the pBLUP schemes. Moreover, the advantage of the genomic over the pBLUP schemes was even more pronounced when the relative weight of trait 2 in the breeding goal increased: ΔFa values were 10 and 36% lower for the least favourable genomic scenario (GE_20%) compared to the most favourable pBLUP scheme for W1:1 and W1:3, respectively.

## Discussion

### Simulation hypothesis

The results presented here were obtained by stochastic simulation of a selected population under several scenarios. Particular attention was paid to modelling a realistic pig population structure with overlapping generations, as well as a genome presenting appropriate linkage disequilibrium and adequate marker density. However, several assumptions and simplifications were necessary, as in any simulation study, mainly to ensure that computation time remained reasonable. For instance, management of inbreeding was not considered in the selection and mating procedures, while this is a major concern for pig breeders. This resulted in markedly higher inbreeding rates and probably stronger selection intensities than what is observed in practice. However, the same selection and mating procedures were applied to both the genomic and the pBLUP scenarios. One can therefore assume that the comparisons were relevant to assess the relative merits of the breeding schemes considered. Nevertheless, the economic results should not be considered as precise predictions but rather as orders of magnitude.

For simplicity, only two unrelated traits were considered in the breeding goal. However, in practice, most traits recorded on phenotyped sibs and candidates are genetically correlated, meaning that measurements on one trait carry information on other traits. On the one hand, multiple-trait pBLUP genetic evaluations are routinely used in current pig breeding schemes to take advantage of this additional information, providing increased accuracies compared to single-trait PEBV. On the other hand, multiple-trait genomic evaluation models have been little explored and evaluated to date, although several models have been proposed. Calus and Veerkamp [15] and Jia and Jannink [16] showed by simulations that multiple-trait genomic evaluations can generate higher accuracies of GEBV than single-trait models. Similar to traditional pBLUP evaluations, greater improvements in accuracy were observed for low-heritable traits that are highly correlated with high-heritable traits. Moreover, according to [15], using multiple-trait genomic evaluations would be particularly advantageous for traits for which the phenotype is not directly observed on candidates, like trait 2 in the present study. However, for such traits, the relative increase in accuracy generated by multiple-trait compared to single-trait models tended to be lower with genomic evaluations than with pBLUP evaluations [15]. This suggests that the superiority of the pig genomic breeding schemes over traditional breeding schemes could be somewhat lower than estimated here for independent traits when traits are correlated and multiple-trait evaluations are used. In addition, genomic selection generates greater genetic gain than pedigree-based genetic evaluations, and could therefore result in fixing favourable alleles of the QTL more rapidly. One can thus assume that genomic selection could change genetic correlations between traits more markedly than traditional selection, modifying the long-term efficiency of the breeding scheme.

Several studies [17-19] have shown that the accuracy of genomic predictions may depend significantly on the number of QTL affecting the traits and on the distribution of their effects. It would therefore be interesting to test the sensitivity of our results by repeating our simulations with different genetic architectures. However, similar relative improvements in terms of average annual genetic trends were observed in [7] for three different QTL densities (10 QTL/M, 30 QTL/M and 60 QTL/M) when changing from BL_ref to GE_ref. Moreover, Daetwyler et al [18] observed that the accuracy of the GEBV calculated with the GBLUP method, as in our study, seemed not to be affected by the number of QTL or by the distribution of QTL variance. These findings suggest that our results should be relatively conservative to the genetic architecture hypothesis, but further simulations are needed to confirm this.

### Accuracy and genetic trends

Our results tended to show that replacing traditional pBLUP genetic evaluations by genomic evaluations in a breeding scheme organized around the combined phenotyping of candidates and sibs could result in more improvement in terms of accuracy of EBV, annual genetic trends and inbreeding than increasing the number of sibs phenotyped for trait 2 while retaining traditional evaluations. This superiority of genomic selection in terms of annual response in the breeding goal was due to the fact that the accuracy of EBV increased significantly for both traits compared to the reference traditional scheme. By contrast, under the pBLUP scheme, increasing the number of phenotyped sibs only improved the accuracy of the EBV for trait 2.

In the nucleus population simulated here, only six animals per litter (seven in litters that had a phenotyped sib) were used for genetic purposes, whereas the average number of weaned piglets per litter in the French Piétrain population that was modeled is approximately eight. Therefore, another way to further improve the efficiency of the pBLUP scheme would be to phenotype these unused animals for trait 1, resulting in a higher accuracy of the PEBV for that trait. However, each candidate in the pBLUP scenarios already had an own phenotype for trait 1, along with phenotypes on its two parents, five full-sibs, and several half-sibs. Therefore, the benefits of these additional animals on the accuracy and genetic gain for trait 1 would be very low, and this option was not considered here.

We compared the accuracy of the pBLUP and genomic evaluations through the correlations between the TBV and the PEBV or GEBV of the evaluated animals. This criterion has emerged as the most commonly used metric to assess the prediction accuracy of a genetic evaluation method, since it has a linear relationship with response to selection. Another criterion to evaluate the merits of different breeding value prediction methods is their ability to produce unbiased estimates of true breeding values. Unbiasedness is a desirable property in genetic evaluation because it enables, in particular, a proper comparison of the genetic merit of animals present in different environments or times, and correct estimates of realized genetic trends. In simulations, unbiasedness of a given breeding value prediction method can be assessed by comparing the estimated and the true genetic trends in the simulated population over time, or by estimating the regression coefficient of true on estimated breeding values at each genetic evaluation time. Unfortunately, these criteria were not calculated in this study.

The potential extra gain for the breeding goal produced by the most efficient genomic scheme (GE_ref) compared to the most efficient pBLUP breeding scheme (BL_90%) depended on the relative economic weights of the traits in the breeding goal. When the trait recorded on candidates and the trait recorded only on sibs had comparable economic weights (W1:1), the potential extra gain of a genomic scheme was considerable, since it improved selection for both traits. By contrast, when a large proportion of the breeding goal concerns the trait recorded on phenotyped sibs (as in the W1:3 case), improving accuracy for the trait recorded on candidates was less crucial, and the potential extra gain of a genomic scheme over a traditional scheme was less.

The accuracy of a candidate’s PEBV depends mainly on the availability of an own phenotype and on the number of its closest relatives that have a phenotype. In this study, this number was on average fixed over time for a given pBLUP scenario, as was the corresponding average accuracy of PEBV. In contrast, the accuracy of GEBV increased with the size of the TP. Thus, it could be assumed, if the simulation had been run for more years and if TP1 had grown continuously instead of being limited to candidates from the last four years after year 10 (for computation reasons), that the accuracy of GEBV for both traits would have tended to increase steadily until the oldest animals in the TP had become uninformative. The superiority of the GE_ref over the BL_ref scheme would therefore have increased further over time. Furthermore, in the simulations, the TP and SNP effects were only updated once a year in order to limit computation time. As a result, the accuracy of the GEBV of candidates, and therefore the selection efficiency in the 17 batches between two updates were lower than if the SNP effects had been re-estimated each TS using TP that would have been continuously updated with animals that are closely related to the candidates (e.g. [20-22]). In addition, selection based on a combination of the candidates’ GEBV and their own phenotypes, which were routinely recorded, would also have slightly increased the accuracy of genomic selection. Thus, our results probably underestimated the potential technical superiority of genomic versus pBLUP schemes.

### Inbreeding

Our results also showed that replacing pBLUP by genomic evaluations in a pig nucleus would have a very beneficial impact on the evolution of population inbreeding, confirming theoretical expectations [23]. The reason is that the breeding value of an individual can be considered as a combination of the breeding value of its parents and a Mendelian sampling term. The Mendelian sampling term is poorly estimated using pBLUP methodology for animals without phenotyped offspring, since the phenotypes of ancestors and relatives mostly contribute to the estimation of the parental component of an animal’s breeding value. As a consequence, the PEBV of related animals are correlated, especially for traits for which the candidates do not have an own phenotype. Consequently, the pBLUP scenario favoured the co-selection of related animals from among the candidates. This phenomenon was even more pronounced for breeding goal W1:3 than for W1:1, because of the greater weight on trait 2, for which full-sib candidates had the same relatives phenotyped for this trait and therefore the same PEBV. In contrast, genomic evaluation results in a more precise estimate of the Mendelian component of the breeding values, producing less correlated GEBV for related animals, less co-selection of related animals, and therefore a smaller increase in inbreeding.

Increasing the number of litters with one sib phenotyped for trait 2 from 10% to 70% also reduced the increase in inbreeding under the pBLUP scenarios when a large weight was given to trait 2 in the breeding goal (W1:3). However, this favourable effect seemed to disappear, or even reverse, when this number exceeded 70%. The favourable trend observed up to 70% was not due to a reduction in the within-litter correlation between the EBV of candidates, as in the genomic schemes, but the result of a change in the heterogeneity of the accuracy of the PEBV for trait 2 among the candidates. In schemes with the smallest numbers of phenotyped sibs, only a few candidates benefited from one full-sib being phenotyped for trait 2. These animals had more accurate, and therefore less regressed, PEBV for this trait compared to the majority of candidates, which had only more distant phenotyped relatives. The animals born in these few litters consequently had a greater chance of being selected to renew the nucleus, especially since their birth litter had a high parental average PEBV. Moreover, the PEBV of litter-mate candidates were highly correlated, since they had the same PEBV for trait 2 (based on the data of the same phenotyped sibs), which had a greater weight than trait 1 in the W1:3 breeding goal. This resulted in high inbreeding rates. Thus, increasing the proportion of litters with a phenotyped sib allowed candidates from more litters to have accurate PEBV for trait 2, which led to the selection of animals from a larger number of families and a deceleration in the increase in inbreeding. However, the procedure applied to choose litters with a phenotyped sib gave priority to litters produced by boars with the fewest offspring phenotyped for trait 2. In other words, the additional 20% phenotyped sibs in the BL_90% scenario compared to the BL_70% scenario mostly came from boars that had had the longest productive life and already a large number of offspring. One can assume that the litter-mates of these phenotyped sibs had consequently more accurate PEBV for trait 2 than the other candidates, and a greater chance to be selected, re-accelerating the increase in inbreeding in the population. In contrast, greater selection pressure was focused on trait 1 in the W1:1 goal, since the accuracy of the PEBV for this trait was higher than for trait 2. Because each candidate had its own phenotype for trait 1, the within-litter correlation between the PEBV was weaker for goal W1:1 than for W1:3, and the co-selection of full-sib candidates was less important. Thus, the level of inbreeding was lower for the W1:1 breeding goal, and the impact of the number of phenotyped sibs on this parameter was not noticeable.

### Extra costs

The benefits of GS in terms of greater annual gain for the breeding goal and lower rates of inbreeding had a cost, since the animals making up the initial TP, as well as 13 770 candidates and 270 phenotyped sibs per year under the reference scheme had to be genotyped. Genotyping all these animals with the porcineSNP60 beadchip would generate very high extra costs, exceeding €2 million per year (considering an approximate current price of €150/animal) for the simulated population considered under the reference scheme. Thus, we assumed that the use of imputation techniques was possible, enabling a dramatic reduction in the cost of implementing GS. Imputation is already routinely used in dairy cattle, with excellent results [12-14]. In pigs, recent studies [24-26] have also shown promising results. For simplicity, we assumed here that imputation of the candidates’ missing genotypes did not cause a loss in selection efficiency, which is not fully realistic and tends to favour the genomic scenarios in the economic comparisons. However, the results of Hickey et al. [12] in pigs suggest that, assuming that candidates are genotyped for 5000 markers or more and have both their parents genotyped with the porcineSNP60 beadchip, the imputation error rate and therefore the loss of selection efficiency would indeed be very low, in which case our results remain valid.

The second solution investigated here was to reduce the implementation costs of genomic evaluation by reducing the number of animals genotyped. This was achieved by downsizing the initial TP1 and reducing the annual number of candidates. In order to limit the loss in selection efficiency, the parental EBV of the litters were used as prior information to detect the most useful animals to genotype. Our results showed that this strategy was effective. The loss of efficiency in terms of response in the breeding goal and rate of inbreeding compared to the best potential genomic scheme (GE_ref) was indeed limited with reductions up to approximately 50% in the initial size of TP1 and in the annual number of genotyped candidates. The limited degree of loss in accuracy of GEBV despite significant reductions in the number of genotyped animals was due to the characteristics of TP1. First, the accuracy of GS is positively related to the size of the TP (e.g. [10,27]) but the marginal increase in the predictive ability of a TP due to the inclusion of additional individuals decreases for large TP [10,28]. In our simulations, TP1 varied from 13 770 in year 6 to 55 080 after year 9 under GE_ref. With such numbers, and given the heritability of trait 1 considered here (h^2^ = 0.4), the impact of a 20% or 40% reduction in size of the TP on accuracy was limited. It is however likely that the loss of accuracy would have been more marked for a less heritable trait. In addition, the accuracy of GEBV is also related to the degree of relationships between the candidates and the TP [29], which was high in the present case since TP1 consisted of candidates, some of which became parents of the next generation. Thus, despite a reduction in size, the candidates still had closely related animals in TP1, which probably limited the loss in accuracy.

The reduction in the number of candidates genotyped also resulted in a reduction in response for trait 1 because of a reduction in selection intensity, since a fixed number of young animals (10 females per TS for each herd, and nine males per TS) were selected from a smaller number of genotyped candidates. However, the loss in selection intensity was lower than might have been expected considering only the decrease in the number of candidates, since the discarded litters were selected based on their parental EBV. Thus, the chances of detecting one of the best candidates from among the animals born in the X% worst litters would have been low anyway, especially for low values of X. To verify this, we repeated the GE_40% scenario simulations for the W1:1 goal but discarding 60% of the litters at random. As expected, the loss in response for trait 1 compared to GE_ref was greater (-24%) than when the litters’ parental EBV was used (−12%). The moderate loss in selection response despite a marked reduction in the number of genotyped candidates observed in this study was consistent with the conclusions reached by Henryon et al. [30] who also showed by simulation that most of the benefits from GS in a pig nucleus breeding scheme, in terms of genetic gain, can be achieved by genotyping a small proportion of the available candidates selected based on their PEBV, including their own phenotype.

In our study, the prior information considered to target candidates to be genotyped was the parental EBV at birth, and piglets from the discarded litters were excluded from the pool of candidates, resulting in lower selection intensity. Another strategy would have been to phenotype for trait 1 also the piglets born in discarded litters and then jointly evaluate the genotyped and non-genotyped candidates using a 1-step GBLUP evaluation method [31,32]. This strategy is similar to that considered in [30] but with different prior information, and might have been even more efficient than the genomic scenarios that we applied, particularly in cases with a small number of genotyped candidates. First, the total number of candidates would have remained unchanged across the compared genomic scenarios, thus limiting the unfavourable impact on selection intensity of genotyping only a proportion of the candidates. Second, the genotyped animals would have had slightly more accurate GEBV for trait 1, resulting from additional records on non-genotyped related candidates. In that context, optimizing the proportion of male-to-female genotyping would be one way to further improve the efficiency of the genomic scenario [30]. The 1-step GBLUP strategy was, however, computationally too costly to be implemented in our simulations, and its additional benefits compared to the genomic scenarios simulated were therefore not evaluated.

At present, the cost of genotyping breeding animals with the porcine60SNP beadchip and of genotyping young animals for sufficient SNPs to minimize the imputation error rate (5000 or more [12]) is similar to the most expensive of our three genotyping costs scenarios. In that context, an additional annual expense of €350 000 to €530 000 (depending on the cost of phenotyping additional sibs and on the relative weights of the traits in the breeding goal) would be necessary to render it more efficient, in terms of genetic gains, to implement genomic evaluation rather than improving the current pBLUP design. Thus, the threshold for implementation of GS is high, but probable reductions in genotyping costs in the future will gradually lower it. In addition, the results obtained by Huang et al. [24] suggest that it might be possible to genotype candidates and phenotyped sibs for only a few hundred markers, while preserving most of the efficiency of genomic evaluations and reducing individual genotyping costs to approximately $25. The use of such optimized imputation strategies would lower the threshold for implementation of GS.

### Extension to other populations

Our results were obtained by simulating a pig nucleus of 1050 breeding females and 50 boars, selected to improve a combination of two unrelated traits. The average accuracy of the PEBV of candidates is expected to be similar in populations of different sizes but with the same structure as simulated here, since a candidate would have approximately the same number of closely related phenotyped animals, regardless of population size. As a consequence, ignoring the effects of population size on selection intensity and the effects of inbreeding, a given pBLUP scenario should provide comparable genetic gains in either a smaller or larger nucleus. In contrast, the sizes of the TP in a genomic scheme with the same structure as here, which are composed of candidates or phenotyped sibs, would increase with size of the nucleus population. Therefore, larger or smaller selection nuclei than simulated here can be expected to result in higher or lower average accuracies of GEBV and realized genetic gains. The relative benefits of the different pBLUP and genomic scenarios considered here therefore depend, to some extent, on the size of the nucleus population. Our proposed methodology must be repeated in order to produce results relevant to the different populations being considered for implementation of genomic evaluations. Further work is also necessary to examine the benefits of GS with respect to more realistic breeding goals that combine several correlated traits.

## Conclusions

Our results show that genomic selection could substantially increase genetic gains achieved in a purebred pig sire line breeding scheme organized around combined phenotyping of candidates and sibs with pBLUP evaluations, while significantly reducing the annual increase in inbreeding in the population. The potential improvements provided by implementing genomic evaluations significantly exceed the possible benefits of increasing phenotyping capacities for the most limited traits while retaining traditional pBLUP evaluations. Implementing a genomic breeding scheme would generate large additional costs. However, substantial savings could be achieved by pre-selecting the candidates to be genotyped based on their parental EBV, and still maintain an advantage over the traditional pBLUP scheme. From an economic point of view, implementing genomic evaluations is not always the most efficient investment to improve the efficiency of a breeding scheme, but a threshold for additional annual expenses can be determined, below which increasing the number of phenotyped sibs while maintaining pBLUP evaluations is preferred, and above which genotyping animals to implement genomic evaluations is more efficient. This threshold depends on the cost of phenotyping additional sibs, on genotyping costs in combination with the imputation strategy, on the breeding goal and on the size of the nucleus population. Therefore, a breeding company must consider the relevance of implementing genomic evaluations based on the characteristics of the population and its possible annual financial commitment.

## Competing interests

The authors declare that they have no competing interests.

## Authors’ contributions

TT designed the simulation study, wrote the computer programs, ran the simulations, interpreted the results and wrote the article. FPH approved the methods used and the simulated scenarios, contributed to the interpretation of the results and critically revised the manuscript. CL approved the methods used and the simulated scenarios, and critically revised the manuscript. All authors read and approved the final manuscript.
